# A reflective display based on the electro-microfluidic assembly of particles within suppressed water-in-oil droplet array

**DOI:** 10.1038/s41377-023-01333-w

**Published:** 2023-12-05

**Authors:** Shitao Shen, Haoqiang Feng, Yueming Deng, Shuting Xie, Zichuan Yi, Mingliang Jin, Guofu Zhou, Paul Mulvaney, Lingling Shui

**Affiliations:** 1 Guangdong Basic Research Center of Excellence for Structure and Fundamental Interactions of Matter, Guangdong Provincial Key Laboratory of Nanophotonic Functional Materials and Devices, School of Information and Optoelectronic Science and Engineering, South China Normal University, 510006 Guangzhou, China; 2 International Joint Laboratory of Optofluidic Technology and System (LOTS), National Center for International Research on Green Optoelectronics, South China Academy of Advanced Optoelectronics, South China Normal University, 510006 Guangzhou, People’s Republic of China; 3 School of Electronic Information, University of Electronic Science and Technology of China, Zhongshan Institute, 528402 Zhongshan, China; 4 ARC Centre of Excellence in Exciton Science, School of Chemistry, University of Melbourne, Parkville, VIC 3010 Australia

**Keywords:** Nanoparticles, Displays

## Abstract

Reflective displays have stimulated considerable interest because of their friendly readability and low energy consumption. Herein, we develop a reflective display technique via an electro-microfluidic assembly of particles (eMAP) strategy whereby colored particles assemble into annular and planar structures inside a dyed water droplet to create “open” and “closed” states of a display pixel. Water-in-oil droplets are compressed within microwells to form a pixel array. The particles dispersed in droplets are driven by deformation-strengthened dielectrophoretic force to achieve fast and reversible motion and assemble into multiple structures. This eMAP based device can display designed information in three primary colors with ≥170° viewing angle, ~0.14 s switching time, and bistability with an optimized material system. This proposed technique demonstrates the basis of a high-performance and energy-saving reflective display, and the display speed and color quality could be further improved by structure and material optimization; exhibiting a potential reflective display technology.

## Abstract (web summary)

We report a reflective display technique using electro-microfluidic assembly of particles based on the dielectrophoretic effect facilitated with droplet compression, demonstrating multi-color display performance with large viewing angle and semi-bistability.

## Graphical abstract

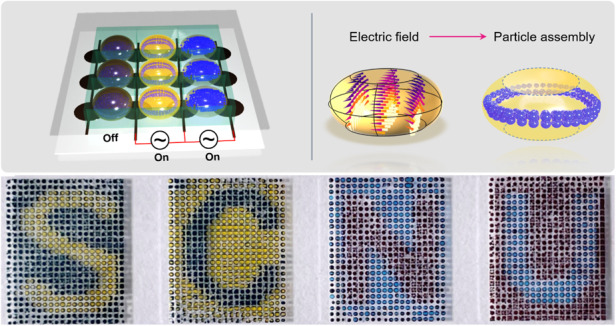

## Introduction

With the rapid development of the Internet of Things (IoTs), large amounts of information need to be displayed to people under proper environmental conditions. Transmissive and emissive displays such as liquid crystal display (LCD) and organic light emission diode (OLED), show high-quality display performance and have been widely applied in electronic terminals^1–4^. Reflective display exhibits comfortable readability and clear vision in outdoor strong light environments with low energy consumption, providing preferential options for electrical shelf labels, E-readers, smart windows, and even applications in buildings and megamalls^5,6^. In the last three decades, several reflective display technologies have been developed, for instance, electrophoretic display (EPD), Interferometric modulator display (IMOD), electrowetting display (EWD), cholesterol liquid crystal display, and photonic crystal display^7–9^.

EPD has been the most successful one to date and has been commercialized and widely applied for e-readers and e-labels^10,11^. It displays information by reflecting the environmental light via colored particles driven by the electrophoretic force to move up and down within pixels (e.g., capsules or wells), achieving paper-like display performance, which is also named E-paper^12,13^. The switching time of a white and black EPD is typically about several hundred milliseconds, while multi-color EPD takes several to tens of seconds to achieve full switching^14,15^. Colored particles with different surface charges are driven by diverse voltage signals, increasing the response time and the fabrication cost. The above issues restrict its application in scenarios requiring a fast response to achieve an excellent interactive experience.

IMOD is based on the interference of reflected light by tuning a flexible thin-film mirror, which displays multiple colors with high switching speed on the order of tens of microseconds; however, its commercialization has been suspended due to its production yield and high-cost manufacture^16,17^. EWD can display video content at a speed of tens of microseconds with multiple colors^18^, while bistability and lifetime still need to be improved^19^. The structural color from photonic crystals has also been applied to display devices^20^ and has been vigorously promoted in recent years^21–23^, but the inherent angle-dependent structural color causes limited viewing angles and the color gamut is still not satisfactory. Overall, it is still challenging to produce a high-quality reflective display with proper switching speed^13^, color^24^, bistability^19^, and viewing angle^25^.

In this work, we report on a potential reflective display technique based on an electro-microfluidic assembly of particles (eMAP) strategy, offering the advantages of easy fabrication, fast response, and multi-color display performance. The colored particles suspended in a water-in-oil droplet are driven to form multiple structures, resulting in a reversible pixel-switching performance in a controllable manner, according to a strengthened dielectrophoretic effect. The colored particles in a water-in-oil droplet can be driven to slide along the curved water-oil interface to assemble at the bottom or top area to form a planar structure and around the equator to form an annular structure in a continuous way, generating closing and opening states and showing multiple blended colors. This is in principle different from the particle motion in EPD.

The optimized eMAP display (eMAPD) can display multiple colors by driving one group of single-color particles into various assembled structures within a dyed droplet. This allows operation in two distinct ways, which we term “light reflection” and “light transmission” modes. The single particle system greatly simplifies the driving system^26^ and increases the response speed of the display^27^. The primary colors of CMYK are created to validate the feasibility and full-color performance. Consequently, the switching between the highest and lowest reflectance states takes about 0.1 s. Moreover, the droplet confinement features a large viewing angle of ≥170°. We have also investigated bistability by matching the particle and water density, maintaining the displayed image for 30 min after the power-off. In addition, the fluidic emulsion system offers a smooth and flexible interface for both encapsulating and manipulating particles, and meanwhile holding the possibility for preparing a flexible display. We believe that this proposed eMAPD would be potentially developed into high-performance and low-cost electronic paper devices.

## Results

### Working principle of eMAPD

**Fig. 1:**
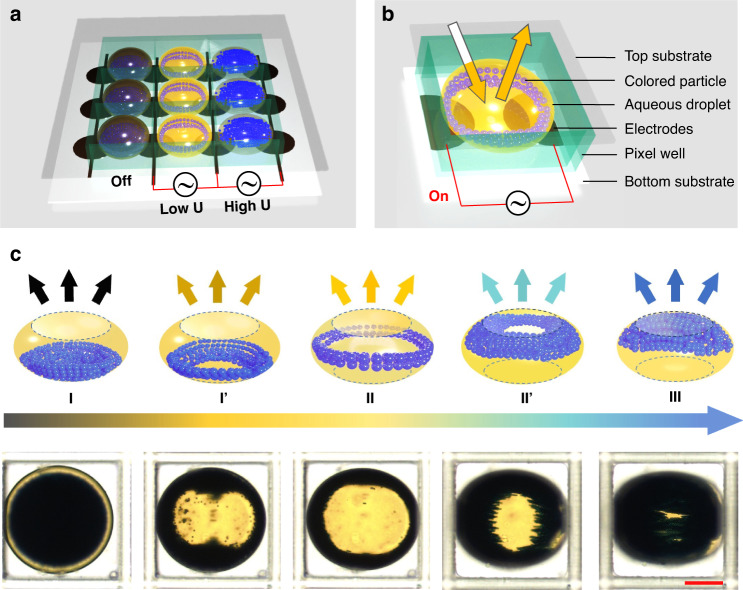
Schematic and experimental results of the electro-microfluidic assembly of particles display (eMAPD). **a** 3D schematic of the eMAPD working principle. **b** Schematic of the detailed device structures and materials of a pixel. **c** Drawings (top row) and experimental photographs (bottom row) of the representative states of a display pixel. States I, II, and III denote the three main states of the particle distribution in a water droplet sitting on the floor, surrounding at the equator, and floating on the ceiling. States I′ and II′ correspond to the representative intermediate transition states from States I to II and from States II to III, respectively. Optical images were taken under a light transmission mode. Scale bar, 100 μm

Dielectrophoretic forces can be generated on particles in a nonuniform electric field^28^. This can be used to control particle motion and distribution, depending on both the applied electric field intensity and gradient. The water phase is used to facilitate the dielectrophoretic effect because of its high polarizability. Here, we take advantage of water-in-oil droplets for micro-confinement, providing a soft and mobile fluidic interface for physical encapsulation and electrical manipulation of colloidal particles. Figure 1 illustrates the working principle and representative results of an eMAPD. A schematic pixel array of the proposed eMAPD is illustrated in Fig. 1a, each water droplet is physically paired in a microwell as a pixel which can display different colors and greyscales by varying the particles and applied voltages. As schematically shown in Fig. 1b, from bottom to top, a display pixel consists of a substrate (typically a reflector), an electrode pair, a hydrophobic dielectric layer, a dyed water droplet containing colored particles, a surrounding oil medium, and a transparent top glass.

In the eMAP, a fluoropolymer film is coated on the electrode to serve as a dielectric layer to protect the semi-elliptical electrodes from electrolysis and reduce the flow fields generated within the droplet due to electroosmosis or electrothermal effects, and meanwhile create a hydrophobic surface for directing the shape of the water droplet^29^. All pixel walls including the top and bottom surfaces are hydrophobic, ensuring the water droplets to contact but do not spread onto the walls. When an AC voltage is applied to the two neighboring electrodes, the generated electric field penetrates across the dielectric film and the oil-water interface into the water droplet, driving the particles to move and assemble according to the dielectrophoretic force. Each water droplet localizes in a cuboid pixel, exhibiting the aspherical (sessile or drum-like) shapes depending on its size and the applied electric field. The electro-microfluidic induced particle assembly then enables the pixel to display blend colors which can be viewed in either reflection or transmission modes.

It is noted that, in this work, the combination of a conductive water droplet phase and an aspherical droplet shape are the key factors enabling the dielectrophoretic-driven particle motion and distribution within a reasonable electric field range. The water droplet works as the confined medium space for particle suspension, encapsulation, and motion. Because of its higher permittivity and conductivity than the oil medium, the droplet is automatically positioned in the gap between the neighboring electrodes under the electric field^30^. As a result, a remarkably consistent and symmetrical electric field distribution within each droplet can be achieved, which ensures that the particle polarization and motion occur in a synchronized manner throughout the droplet array. Silicone oil is selected to preserve the electrohydrodynamic effect and avoid water evaporation. Such a water-in-oil emulsion system establishes an inherent barrier to avoid ink leakage and cross-contamination among adjacent pixels. In addition, the oil-water fluidic interface enables flexible manipulation of droplet shapes to comfort the pixels and meanwhile provides a flexible and suitable fluidic interface for particle motion and assembly. In this way, the optical light reflection and transmission can be well-tuned via the applied electric field combined with particle and droplet properties, achieving a single light valve and arrayed display performance.

In the pixel unit of such an eMAPD, multiple and reversible states have been observed, as depicted in Fig. 1c, which can be defined as States I, I′, II, II′, and III. Hereby, the States I′ and II′ represent the transitions between the two adjacent states, i.e., I-to-II and II-to-III, respectively. When a voltage is applied via the paired electrodes, the particles start to assemble along the electric field lines. With an increase in the applied voltage, the particles gradually rise and conduct to diverse states, presenting different colors and hues. In the initial State I, the incident light goes through the dyed water droplet and then is reflected by the particles sitting on the droplet floor, corresponding to a blended color of droplet and particles. Once particles reach the droplet equator, they form an annular structure denoted as State II, presenting an “open” state in which the incident light passes through the transparent droplet and then is reflected by the substrate. Hereby, the eMAPD displays the blended color of the water droplet and the underneath substrate. When the particles are driven to assemble at the droplet ceiling, namely State III, the incident light is reflected directly by the particles, and creates a reflection state with solely the particle color. Between States I and II, and States II and III, there are multiple intermediate states. Hereby, we define States I′ and II′ to represent two typical transition states, showing how the particles gradually move and distribute within the droplet in a continuous way. These states and controllability are essential for realizing multiple colors or multi-level grayscale in an eMAPD.

The dielectrophoretic (DEP) force acting on a colloidal particle suspended in a liquid medium can be written as^31^,

$$\left\langle {{\bf{F}}}_{\text{DEP}}\right\rangle =2{\pi \varepsilon }_{\text{m}}{\text{Re}}\left[{f}_{\text{CM}}\right]{R}^{3}\nabla {|{{\bf{E}}}_{\text{rms}}|}^{2}$$

where **E**_rms_ is the root mean square (rms) of the electric field. For a sine wave, the rms value is ~0.707 times the peak value. ∇|**E**_rms_|^2^ represents the gradient of |**E**_rms_| squared, *ε*_m_ is the permittivity of the liquid medium, and *R* is the particle radius. Re[*f*_CM_] represents the real part of the Clausius-Mossotti (CM) factor (*f*_CM_), meaning the effective polarizability of a particle described as,

$${f}_{\text{CM}}=\left(\frac{{\widetilde{\varepsilon }}_{{\rm{p}}}-{\widetilde{\varepsilon }}_{{\rm{m}}}}{{\widetilde{\varepsilon }}_{{\rm{p}}}+2{\widetilde{\varepsilon }}_{{\rm{m}}}}\right)$$

where ${\widetilde{\varepsilon }}_{{\rm{p}}}$ and ${\widetilde{\varepsilon }}_{{\rm{m}}}$ indicate the complex permittivity of the particle and the medium, respectively.

In this work, the colored polystyrene (PS) particles with lower permittivity and conductivity than the water phase are used as the reflective materials, which are subjected to negative dielectrophoretic (nDEP) forces and flow upwards in the droplet. The water droplets are subjected to positive DEP (pDEP) forces due to their relatively high permittivity and conductivity compared to the oil medium. The paired water droplet in a pixel is therefore attracted into the gap between a pair of semi-elliptical electrodes with high electric field strength; meanwhile, it undergoes dielectrowetting effect under an AC field^32^.

When an AC voltage is applied, the electric field enters the oil phase from the electrode surface and then penetrates across the oil-water interface into the water droplet. The electric potential in the oil phase (*U*_oil_) falls rapidly due to its relatively low permittivity compared to the water phase^33^. It is worth mentioning that the droplet shape strongly affects the displacement (*γ*) that the field crosses through the oil phase, and thus affects *U*_oil_ (*U*_oil_ = -**E·***γ*). As *U*_oil_ is negatively correlated with the potential drop in the water droplet, a low *γ* is preferable to obtain a high *E* distribution in the water droplet. Dielectrowetting induces an increase in the droplet-substrate contact area and a decrease in *γ*, and therefore facilitates the particle assembly and motion. More importantly, the height and length of the pixel walls determine the shape of the corresponding droplets. The relatively large and low height-to-length ratios correspond to the sessile and drum-like droplet shapes, respectively. In comparison to a sessile shape, a drum-like droplet shows a large droplet-substrate contact area and can therefore enhance the internal electric fields within a water droplet. In the aspect of display performance, the pixel height and length are also related to the displaying color and resolution.

**Fig. 2:**
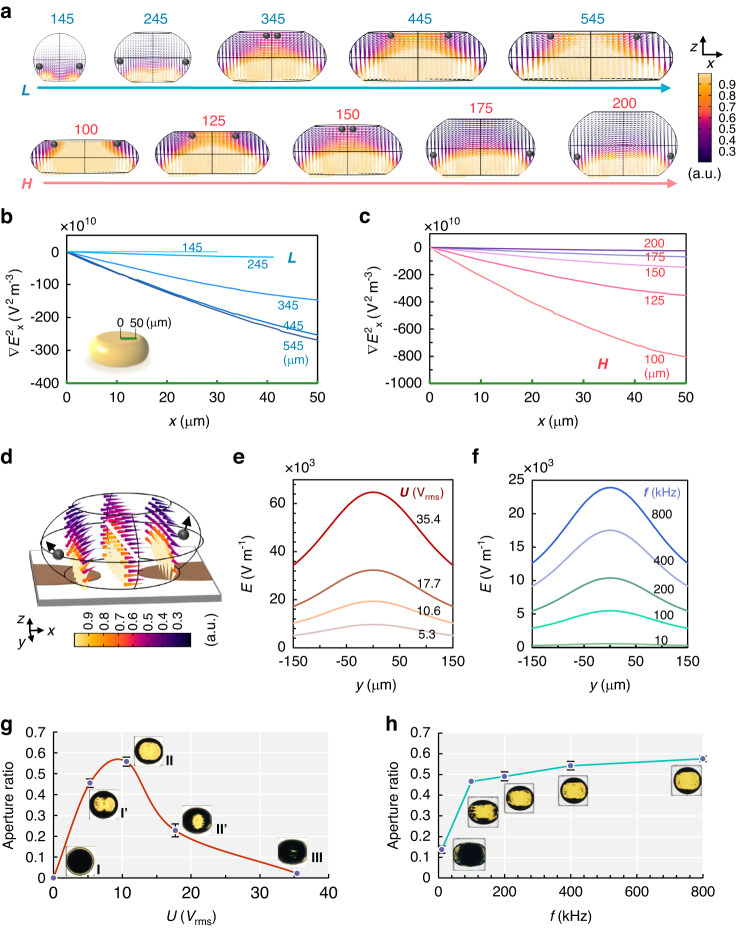
Optimization of the droplet pixel. **a** Electric field distributions inside droplets with various side lengths (*L*) of 145, 245, 345, 445, and 545 μm at a fixed height (*H*) of 145 μm, and for various *H* of 100, 125, 150, 175, and 200 μm at a fixed *L* of 345 μm. **b** *x*-component of ∇|**E**_rms_|^2^ (∇*E*^2^_x_) distribution along the radial line from the center to the right edge of the droplet (marked in green) at different *L*. **c** ∇*E*^2^_x_ distribution along the radial line from the center to the right edge of the droplet at different *H*. The green line is 10 μm away from the droplet’s top. **d** Simulated electric field distribution in a conductive 3D water droplet surrounded by an insulating oil. The red line indicates the central line across the droplet along the *y*-axis. Electric field distribution along the white line at different **e** applied voltage and **f** frequency corresponding to the image in (**d**). The States I’, II, II’, III in **e** represent the results obtained at 5.3, 10.6, 17.7, and 35.4 V_rms_, respectively, at *f* of 400 kHz. The aperture ratio varies with *U* (**g**) and *f* (**h**). The electric field of 10.6 V_rms_ and 400 kHz are applied in the simulation in (**a**–**d**). *L* = 345 μm and *H* = 150 μm are applied in (**d**–**h**)

To investigate the mechanism of such an electrical responsive system, both theoretical and experimental characterizations have been carried out, as presented in Figs. 2, S1, and S2. The electric field distribution was simulated using COMSOL 5.5a. We assume that the water droplet is tangential to the surrounding pixel walls. In Fig. 2a–c, the droplet geometries corresponding to the pixel structures are demonstrated. We abbreviate the rms value of electric field strength (|**E**_rms_ | ) as *E*. At the same pixel height (*H*), with the increase of the side length (*L*), the droplet is compressed from spherical to drum-like shapes. Consequently, the electric field strength (*E*) within the droplet increases (Fig. 2a), and thereby the particle motion can be accelerated. In addition, the longer side length corresponds to the larger droplet’s occupying area (*O*_droplet_) as viewed from the top, which can be calculated as:

$${O}_{\text{droplet}}=\frac{\pi {L}^{2}}{{4\left(L+T\right)}^{2}}$$

where the pixel wall thickness (*T*) is fixed at 20 μm to maintain its mechanical strength. A larger *L* is correlated to the higher contrast and saturation of the colors but yields a lower resolution for a display device. However, excessive increases in *L* will hinder the particles from reaching the ceiling center of the droplet, which is unfavorable for the State III transition, as presented in Fig. 2a. The reason lies in the fact that the particles subjected to nDEP typically prefer to escape from the high *E* region (yellow) and stay in the low *E* region (purple). The field distribution in the droplet with *L* = 445 and 545 μm shows a trapezoidal high *E* region that overlaps with the droplet ceiling center, keeping particles from reaching the center. To evaluate this nDEP effect, we simulated the *x* component of $\nabla {\text{|}{\bf{E}}}_{\text{rms}}{\text{|}}^{2}$, denoted as ∇*E*^2^_x_, distributed near the ceiling center at a fixed value of *H* = 150 μm, accounting for *F*_DEP_ ∝ ∇*E*^2^. As presented in Fig. 2b, the decrease in ∇*E*^2^_x_ from the center towards the edge of a droplet ceiling indicates that the particles experience the repulsive forces from the ceiling center. We experimentally found that *L* = 345 μm (*H* = 150 μm) is the threshold value to ensure that the potential energy of electrical dipole-dipole interactions (Fig. S2) can overcome the *x* component of the nDEP effect, allowing particles to reach the droplet ceiling center. The higher *L* corresponds to a stronger nDEP effect, hindering the particles from assembling into State III. Therefore, the moderate value of 345 μm is selected as the optimized side length.

As shown in Fig. 2a, at fixed *L* = 345 μm, *E* within the droplet reduces with the increase of *H*. The State III transition is unreachable at *H* = 100 μm, and the low *H*/*L* value pushes the ceiling close to the electrode gap. The *E* value at the ceiling center is much higher than that of the ceiling edge, hindering the particles from reaching the top center of the droplet. In addition, the nDEP effect is also evaluated as shown in Fig. 2c. As a result, an increase in *H* assists the system in reaching State III. Collectively, a moderate value of *H* is preferred. Hereby, *H* = 150 μm has been selected corresponding to *L* = 345 μm.

Using these optimized parameters, we have conducted a combined simulation and experimental investigation to understand the mechanism of the eMAPD. As seen from Fig. 2d, in the *y*–*z* plane, *E* decreases radially outward from the center towards the droplet surface, inducing the particles to distribute at the low-strength region near the edge of a droplet. The similar *E* distribution in the *x*–*z* plane and *y*–*z* plane jointly leads to the particles radially spreading from the center to the edge and moving upwards along the droplet interface. Therefore, a raised annular structure can be obtained.

The equilibrium position of the particles under the electric field can be tuned by the amplitude and frequency of the applied electric field. At a fixed frequency (*f*) of 400 kHz, as the amplitude of the applied field (*U*) increases from 5.3 to 35.4 V_rms_, the particle assembly structures can gradually change from States I to III. The simulated *E* distributions along the *y*-axis (white line in Fig. 2d) are demonstrated in Fig. 2e. Figure 2f exhibits the *E* distribution along the *y*-axis at various values of the frequency *f* at a fixed value of *U* = 10.6 V_rms_. Since the dyed water droplet is conductive, the electric field is shielded from entering the droplet at low *f*^28^. As *f* increases, the liquid molecular polarization gradually dominates, allowing the electric field to penetrate the droplet, accompanied by an increase in *E*.

As a display technology, the aperture ratio is used as one of the key parameters to quantify the display performance, which is defined as the ratio of the light transmission area to the total coverage area of a droplet, as shown in Fig. 2g. At *U* = 10.6 V_rms_ and *f* = 400 kHz, the particles distributed at the droplet equator form an annular structure corresponding to an aperture ratio of ~56%. When *U* increases to 35.4 V_rms_, the particles reach the droplet ceiling (State III), achieving an aperture ratio of ~2% corresponding to a 98% closing. In addition, as shown in Fig. 2h, the aperture ratio of 13% corresponding to *f* = 10 kHz matches the low *E* value in the simulation (Fig. 2f). As *f* increases from 400 to 800 kHz, the aperture ratio rises slightly with an increase of *E*. Therefore, *f* = 400 kHz has been selected in our experiments.

The contact areas between the droplet and the top and bottom substrates have also been examined and applied in the simulations (Fig. S1a). Figure S1b–d presents the droplet deformation degree varying with *U*. Since the electric field lines distribute mainly along the *x*-axis due to the dipolar effect, most particles assemble into chains along the *x*-axis and perpendicular to the *y*-axis (marked in light blue rings in the bottom inset figures) induced by the difference between *λ*_x_ (the midline length along *x*-axis of the opening area) and *λ*_y_ (the midline length along *y*-axis of the opening area). In addition, within the droplet, the flow field was also simulated to explain the alternative current electrothermal (ACET) flow effect. At State II, the fluid flow velocity is in the order of 1 μm/s which is negligible to the particle assembly. However, as the AC field amplitude increases, the ACET effect might play a role at State III, as shown in Figs. S2 and S3. Therefore, in experiments, we limited the electric field to 35 V_rms_ to avoid the ACET effect and reduce energy consumption. Overall, the light transmission and reflection modes and their ratio are determined by the particle distribution in droplets corresponding to the assembly structures and their position, as presented in Fig. 1. While the assembly processes and state transitions are mainly contributed by the combination of dipolar interactions and dielectrophoretic effects. The assembly structures, which are determined by both the dipolar interactions among particles and the droplet confinement effects. The spatial positioning of the particle assembly structures in a droplet is dominated by the dielectrophoretic forces which are related to the applied electric field and the physicochemical properties of the droplets and particles. The dynamics of structure formation and change processes will govern the display speed and the possible blended colors.

### Characterization of eMAPD

As exhibited above, each droplet unit displays both light shading and transmission in different states generated by the applied electric fields. Figure S4 demonstrates a large visual difference between the OFF and ON appearances, corresponding to the states I and II obtained at 10.6 V_rms_ and 400 kHz. Such a patterned droplet array can be selectively driven either synchronously or asynchronously to achieve a switchable color display. As a result, a display device can be obtained by integrating the droplet units in a large area with high density. An array of droplets (pixels) with a pitch size of 150 μm represents the display resolution of about 70 PPI which is applicable for a large screen application (e.g., 100 inch, 8 K resolution; or 60 inch, 4 K resolution).

The displayed states (colors) of the eMAPD are dependent on the combination of the colored particles, the dyed droplets, and the electrode/substrate/reflector color, as presented in Fig. 1. The blue PS particles (*d* ~ 4 μm) dispersed in dyed yellow aqueous solution with optimized concentration have been used as the droplet materials. The solid content is optimized according to maximized coverage over the pixel at States I and III. The aqueous solution is optimized to satisfy the conductivity and reflectance requirements according to dielectrophoresis and display efficiency (as shown in Fig. S5). Figure S6 presents the reflection spectra of the color particles and dye solutions. The optimized parameters of blue particle concentration of 12 wt%, yellow dye concentration of 0.085 wt%, and droplet diameter (*D*) of ~340 μm were chosen to demonstrate the eMAPD performance in the following experiments.

**Fig. 3:**
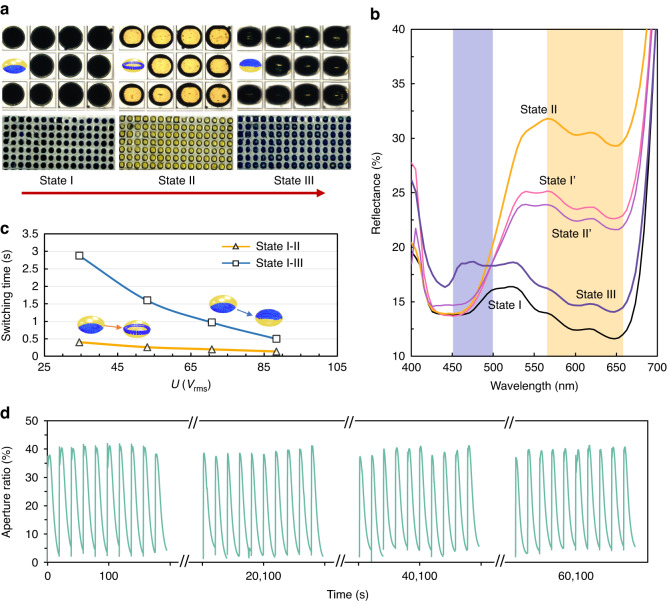
Characterization and optimization of the eMAPD device. **a** Details and overall appearance of the States I, II, and III. **b** Measured reflectance in a visible light range (wavelength, 400–700 nm) for the five States. In (**a**, **b**), the states transform from I to II and then to III and are driven at the applied voltages of 0, 5.3, 10.6, 17.7, and 35.4 V_rms_, respectively, at a fixed frequency of 400 kHz. **c** Switching time versus applied *U* with switching process at State I to II, and State I to III. **d** Repeatability and reliability test of a droplet array. Switching State from I to II under the applied electric field (On) at 35.4 V_rms_ and 400 kHz, and from States II to I achieved by gravity at 0 V_rms_ (Off)

As shown in Fig. 3a, when *U* increases from 0 to 10.6 and 35.4 V_rms_ with *f* = 400 kHz, the displayed colors of the eMAPD gradually change from black to yellow and then dark blue according to the particle assembly states transforming from States I to II and III. At State I, the PS particles sitting at the droplet floor, corresponding to the “black” color which is the combination of the yellow water (incident light passing through) and the blue particles. At State II (*U* = 10.6 V_rms_ and *f* = 400 kHz), the blue PS particles move upwards to the droplet’s equator and assemble to form an annular structure around the droplet interface, allowing the incident light to pass through the droplet and then be reflected from the white reflector, thus showing yellow color. Figure 3b exhibits the measured reflection spectra of the pixels, lying within the corresponding wavelength (*λ*) range of 520–660 nm, and the reflectance at ~571 nm increases to 31.7%. At State III, when most particles arrive at the droplet ceiling at *U* = 35.4 V_rms_ and *f* = 400 kHz, the incident light is directly reflected by the particles, and the droplet exhibits a dark blue color. The corresponding reflection spectrum shows strong reflectivity in the range of 430–480 nm, and the reflectance at ~461 nm is ~18.5%. Furthermore, the transition States I’ and State II’ also show corresponding strong reflection in the displayed color range. The State II’ exhibits higher reflectance at blue wavelengths and lower reflectance at yellow wavelengths than the State I’, corresponding to the vertical position of the assembled particles in the droplet. In this way, a multi-color with multi-level grayscale display performance has been successfully achieved according to the tunable reflective status of States I, I’, II, II’, and III, corresponding to the images in Fig. 1c.

The DEP effect, in principle, can achieve controllable particle motion directions by two strategies. One is to formulate the solid particle and the fluid with proper dielectric properties based on pDEP and nDEP forces. The other is to integrate the top electrode to generate a mirror electric field. The electric field applied on the upper electrodes can induce an nDEP force that theoretically causes the corresponding mirror electro-fluidic performance to drive the particle to quickly move downwards from State II or State III to State I. In this work, we focus on the upwards-switching performance from State I to II and then III, investigating the dynamics of the display performance (Fig. 3c), and the passive downwards switching was achieved by directly turning off the applied electric field to let particles settle back to the droplet floor. In a pixel of 150 × 345 × 345 μm (~70 PPI), the switching time of 0.49 s is achieved and can be further significantly reduced to within 0.05 s in theory by compressing the height of the device to 50 microns, based on the intrinsic feature of dielectrophoresis.

The dielectrophoretic force is a function of the applied electric field, which can be described as *F*_DEP_ ∝ ∇*E*^2^. Therefore, experimentally, we found that the increases of *U* from 34.5 V_rms_ to 88.3 V_rms_ at *f* = 400 kHz, can reduce the switching time (an OFF-ON-OFF process from State I to State III) from 2.88 s to 0.49 s, and the switching time (from State I to State II, OFF-ON) from 0.4 s to 0.14 s, as presented in Fig. 3c. To verify the stability and reliability of this proposed eMAPD, we have also carried out multiple switching tests. The prepared eMAPD shows stable display performance without obvious decay after 3000 cycles of ON-OFF tests at 35.4 V_rms_ and 400 kHz in a normal lab environment without additional control, and the results are presented in Fig. 3d.

### Color

To create a true color reflective display, the primary colors of CMYK (cyan, magenta, yellow, black) are commonly used to demonstrate the performance. The K (black) color can be produced by rationally mixing C, M, and Y with their complementary colors of R, G, and B, respectively. Hereby, we have selected the droplets with C, M, and Y colors, and introduced the particles with corresponding complementary colors to demonstrate the color variation. The eMAPD thereby can perform multiple color combinations of cyan and red, magenta and green, and yellow and blue.

**Fig. 4:**
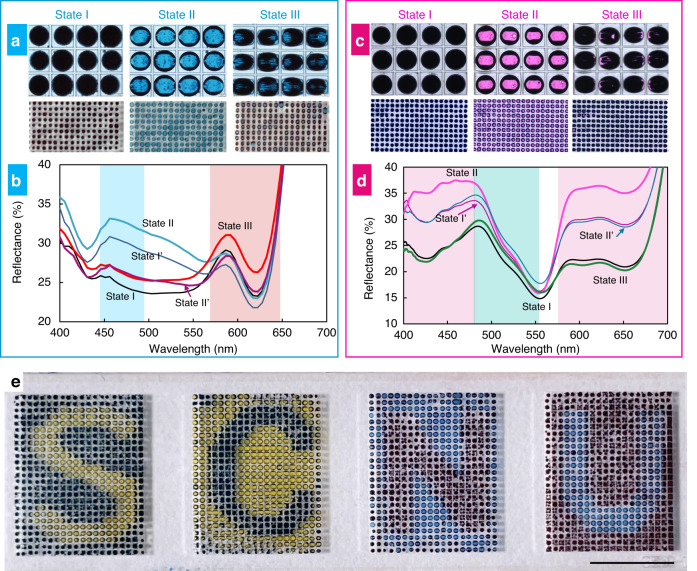
Feasibility of a multiple-color display. **a** Images and **b** reflection spectra at different states for the eMAPD with the cyan droplets containing red particles. The States I, II, and III are obtained at *U* of 0, 14, 42 V_rms_, respectively, at *f* = 400 kHz. **c** Images and **d** reflection spectra at different states for the eMAPD with the magenta droplets containing green particles. The States I, II, and III are obtained at *U* of 0, 10.6, 35.4 V_rms_, respectively, at *f* = 400 kHz. **e** Representative eMAPD panels showing multiple colors and contrasts for “S”, “C”, “N”, and “U” letters based on the yellow-blue and cyan-red system driven at 14 V_rms_ and 400 kHz. Scale bar, 2.5 mm

As presented in Fig. 4a, the cyan, red, and cyan-red colors are achieved by dispersing 12 wt% red PS particles (~5 μm) in a cyan dye solution (malachite green oxalate, 0.045 wt%). By increasing the applied voltage, the dark red, cyan, and red colors are obtained at 0, 14, and 42 V_rms_, respectively, at a fixed frequency of 400 kHz. Figure 4b shows the corresponding reflection spectra of the five states. The two peaks of ~450 nm (blue background) and ~617 nm (red background) in the spectra correspond to the cyan droplets and red particles, respectively. In the initial State I, the combination of the cyan droplet and red particles exhibits a dark red color. A bright cyan is achieved in State II, with the reflectance at the ~476 nm peak reaching 32.5%. When changing to State III, the reflectance at ~617 nm (red) increases to 26.3%, and the droplet exhibits a red color. In addition, during the transformation process, both cyan and red peaks continuously change with the particle assembly states and positions, thus exhibiting various colors and greyscales.

The magenta-green color system has also been investigated, as shown in Fig. 4c, for the States I, II, and III achieved at *U* of 0, 10.6, and 35.4 V_rms_, respectively, with *f* = 400 kHz. Figure 4d exhibits the measured reflection spectra corresponding to the five states of the eMAPD. At the initial State I, the combination of the magenta droplet and green particles exhibits a black color. In State II, a bright magenta color is observed with reflectance values of 34.9% and 36.3% at ~476 nm and ~616 nm, respectively. When changing to State III, a dark green color is observed with the reflectance at ~527 nm (green) increasing to 23.3%, and the saturation of State III can be further improved by increasing the green particle reflectance (Fig. S6).

To demonstrate the practical applicability of the prepared eMAPD, we have made a display panel with four segments filled with yellow droplets containing blue particles and cyan droplets containing red particles. As presented in Fig. 4e, the “S” “C” “N” “U” letters are readily observed when applying an AC electric field of 14 V_rms_ and 400 kHz to the corresponding electrode pairs. The four chromatic letters are the yellow “S” on a blue background, the blue “C” on a yellow background, the red “N” on a blue background, and the blue “U” on a red background. A contrast ratio of about 3:1 has been achieved for all these display panels.

**Fig. 5:**
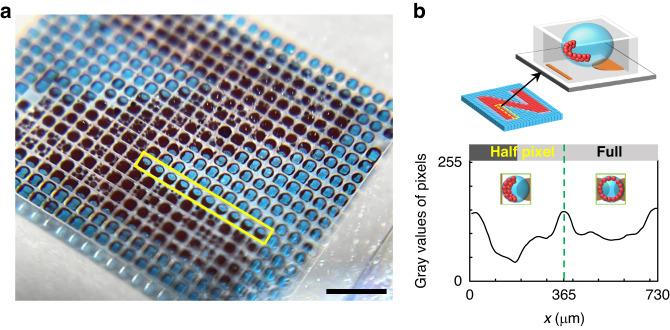
Half-pixel particle assembly phenomenon in eMAPD. **a** Magnified photograph showing the half-pixel particle assembly performance. Scale bar, 1.5 mm. **b** Schematic of the particle assembly in half-pixel with single semi-elliptical electrode pixel design. The gray values of the pixels were acquired from the grayscale image converted from a color image containing two columns of pixels. Each data represents the mean value of the area covered with 9 rows of pixels

In addition, we have found the asymmetrical particle assembly phenomena at one-half of the droplet (half-pixel) corresponding to the predesigned semi-elliptical electrode pair, as depicted in Fig. 5a. A minimal gray value has been achieved with the particles assembling at approximately one-half of the pixel range with *x* in the range of 0–168 μm. Such an asymmetrical particle assembly phenomenon, on the other hand, helps achieve sharp line edges (Fig. 5b), meaning high resolution for display performance. This suggests that the display resolution is not strictly limited by the droplet size and can be improved either by reducing the pixel size or the electrode design combined with the driving strategy. The feasibility of this half-pixel driving scheme is thus highly advantageous for display performance. More importantly, the light transmission feature at State II of this eMAPD can expand the displayable color range via a sandwich structure. This could promise tremendous opportunities because the sandwich structure can achieve multiple colors without reducing the display speed.

### Wide viewing angle

**Fig. 6:**
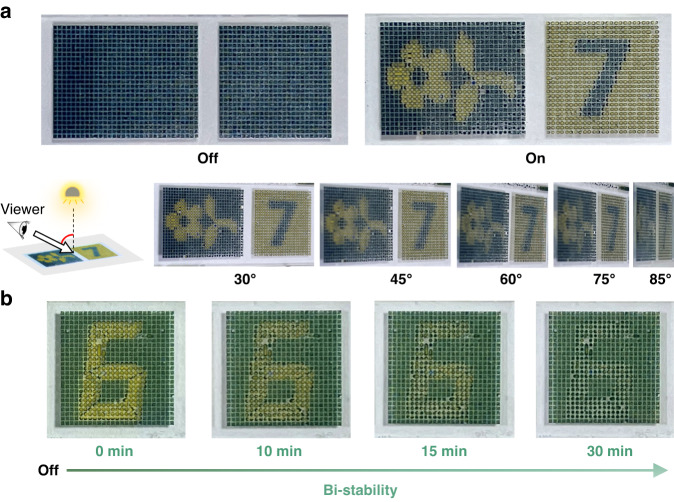
Demonstration of the large viewing angle and bistable display features of the eMAPD. **a** Photographs of the displayed “flower” and “7” patterns taken from different viewing angles. The applied electric field is 10.6 V_rms_ and 400 kHz. **b** Photographs showing the displayed “6” pattern being visible after 0, 5,10, and 30 min of power-off

Viewing angle is also a key factor affecting the visual perception of readers. In a structurally tuned color display, like the liquid crystal display and the photonic crystal display, the viewing angle is limited by the molecular and nanoparticle arrangement. In this proposed eMAPD, the displayed color is from the combination of the colored particles, the dyed water droplets, and the reflector. The three-dimensional droplets guarantee wide viewing angles, as depicted in Fig. 1. Figure 6a shows the patterns of “flower” and “7” based on the yellow-blue system. The displayed information is clearly visible when viewed at a bias angle of 0°–85°, indicating a 170° viewing angle. The evaluation results of optical contrast and luminance at different viewing angles are presented in Fig. S8. As shown in Fig. S8a, the contrast ratio slightly decreases with varying viewing angles, which is consistent with the distinct visual performance of the displayed patterns “flower” and “7” at different viewing angles (Fig. 6). In addition, we evaluated the relative luminance at different viewing angles (Fig. S8b). The results demonstrate that the eMAPD device exhibits a mild luminance attenuation within an acceptable range as the viewing angle varies in the range of 0°–160°.

### Bistable display feature

Bistability is an important prerequisite for reflective displays, which makes it possible to maintain different display states with minimum energy consumption. In addition, this feature offers the capability of storing information without a power supply. Such properties are highly beneficial for applications such as electronic books, electronic labels, and smart windows. Based on the working principle of eMAPD, by balancing the density of the particles and the water phase, semi-bistability has also been achieved. We have developed a density-matching dispersion with PS particles (density, ~1.06 g cm^−3^) suspended in the aqueous solution containing 14.2 wt% glucose and 4.35% v/v glycerol. As depicted in Fig. 6b, the displayed “6” is still visible after 30 min of power-off. The micrometer-size PS particles experience weak Brownian motion and the previous state can be long-term retained after power-off. With further optimization of the material quality, we believe that a highly bistable feature, lasting days, can be achieved with this eMAPD.

## Discussion

We proposed and validated a reflective display based on electro-microfluidic assembly of particles (eMAP) technology. By programmably driving colloid particles to assemble into multiple structures with distinct positions in the aqueous droplet pixel through electric fields, we were able to achieve tunable blend colors and grayscales, and ultimately reflective display function via the ordered droplet array. We demonstrated that the deformation and dielectrowetting featured by oil-in-water droplets play a significant role in the internal electric field distribution, which in turn determines the particle motion and assembly structure. Accordingly, the pixel structure was optimized to direct the droplet shape, allowing fast, multi-state, and reversible particle assembly to enhance display performance. Our eMAPD results highlight the importance of curved interfaces in particle-based technology to achieve high-performance multi-color displays. Compared to traditional electrophoretic display technologies, the light transmission and reflection modes offered by eMAPD provide a promise for the fast switching, low-cost, multi-color reflective display technology. The photolithography microwell fabrication and micro-confinement induced droplet fusion allow us to fabricate uniform and stable droplet pixels in high-density and large areas to satisfy the display application.

The display performance with three primary colors and multiple grayscales has been obtained by driving the color particles inside a complementary dyed water droplet array. Under optimized geometry and materials, the reflective display with ≤0.14 s switching speed, ≥5 colors and gray levels, and ≥170° viewing angle has been achieved. Bistability has also been demonstrated for maintaining displayed information for 30 min after power-off via density matching of the particle and the aqueous droplet.

We believe that our proposed eMAP display technology offers a wide range of advantages, including achieving fast particle assembly at low voltage, employing easily accessible and simple particle systems for sharp multi-color and high contrast displays, while enabling a bistable display via the synergistic matching of selected particle and fluids. The material preparation and the pixel fabrication technology are developed based on the electro-fluidic platform initially developed in our group. Further optimization of the pixel arrangement and droplet array filling techniques to increase the droplet coverage area, combined with the improved display materials, could yield higher-quality display performance. We believe that this proposed eMAP-based display technique is an excellent candidate for reflective displays, potentially for corresponding application areas.

## Materials and methods

### Display devices

Figure 1a, b show a schematic of the device design. The indium-tin-oxide (ITO)-glass slides were thoroughly cleaned sequentially with detergent, deionized water, ethanol, and isopropanol prior to use. The designed electrode patterns, semi-elliptical electrode pairs with 365 μm pitch and gap distance of 60 μm arranged in a row along a short axis of 130 μm, were fabricated using a standard photolithography process in a cleanroom^29^. The hydrophobic insulating layer of Hyflon with a thickness of ~140 nm was formed by spin-coating a Hyflon solution (solid content of 2.5 wt%) on the patterned ITO-glass. Oxygen plasma (Harrick Plasma, PDC-002) was used to modify the Hyflon surface, and then spin-coating SU-8 2075 photoresist (Microchem, Newton, MA) on its surface to form a 140–150 μm thick film. Subsequently, SU-8 grids with a width and length of 345 μm and a gap of 20 μm were fabricated using the second photolithography step. To enclose eMAPD, a glass coverslip was used after filling the SU-8 grids with W/O emulsions. Then AC signals were applied to the ITO electrodes by a connected function generator (AFG 1062, Tektronix, Inc., USA) and an amplifier (ATA-2042, Aigtek Co., Ltd, Xi’an, China).

### Materials

DI water was prepared using an Ultrapure Water System (Water Purifier, Chengdu, Sichuan, China) with an initial resistivity of 18.25 MΩ·cm. Blue (*d*, 4 μm), red (*d*, 5 μm) PS particles were purchased from Aladin (Shanghai, China). Green PS particles (*d*, 4 μm) were purchased from Tianjin Baseline Chromtech Research Center (Tianjin, China). The silicon oil (5 cst) was purchased from Sigma-Aldrich. Dyes of malachite green oxalate, rose bengal, orange IV were purchased from Aladin (Shanghai, China). Poly(ethylene glycol) diacrylate with *M*_n_ ~ 575 was ordered from Sigma-Aldrich (Shanghai, China). Silicon oil with surfactant KF-6017 (0.5% v/v) (ShinEtsu, Japan) was selected as the continuous medium phase. Malachite green oxalate, rose bengal, orange IV were dissolved in DI water at concentrations of 0.045 wt%, 0.1 wt%, and 0.085 wt% to form dyed water phase, and poly(ethylene glycol) diacrylate was dissolved at 0.5 wt% to stabilize particles in aqueous phase. Colored particles with a solid concentration of 12 wt% were dispersed in the dyed water which was then added into the oil phase at a volume ratio of 1:5.

### Droplet array

As shown in Fig. 3a, the integrated eMAP device consists of Teflon coated ITO electrode array and photoresist (Microchem, SU-8 2075) grids with each droplet filled with one aqueous droplet (including particles) and immersed in an oil (dielectric) phase. The mixture of water and oil were mechanically emulsified using a Vortex Genie 2 (Scientific Industries, setting 4) for 10 s, followed by filling the emulsion droplets into the grids via a screen-printing method. Subsequently, an electrofusion method is applied to obtain the monodisperse one-to-one paired droplet in each pixel^34^. A top plate was then applied to seal this device with the grid wall working also as a spacer to form a stable eMAP display device. Figure 4e shows a eMAPD with 2000 pixels (4 × 25 × 20).

### Numerical simulation

COMSOL Multiphysics (Version 5.5) was employed to simulate the E-field distribution in the pixel. The electric potential was assumed to satisfy the Poisson’s equation. For more details on modeling see Supplementary Information.

### Data acquisition and analysis

The dyed droplets, colored particles, and the dynamic assembly processes and structures were observed and captured/recorded using an inverted microscope (IX73, Olympus Co., Tokyo, Japan). The Image J software was used to analyze the aperture ratio and switching speed data contained in optical images and video frames. The overall devices were taken using an iPhone 12 camera. The reflectances of particles, dye solution and eMAPD were measured using a fiber-optic spectrometer (USB2000+, Ocean Optics). As shown in Fig. 5, gray values of pixels are obtained from the image converted to greyscale by using the MATLAB function rgb2gray.

### Supplementary information

Supplementary Information for a reflective display based on the electro-microfluidic assembly of particles within suppressed water-in-oil droplet array

## Data Availability

All data needed to evaluate the conclusions in the paper are present in the paper and/or the Supplementary Materials. Additional data related to this paper may be requested from the authors.
