# Supplementary material for: A reflective display based on the electro-microfluidic assembly of particles within suppressed water-in-oil droplet array
Source: Light Sci Appl. 2023 Dec 5;12:290. doi: 10.1038/s41377-023-01333-w (PMC10697941; doi:10.1038/s41377-023-01333-w)
Supplement: Supplementary file 1 — Supplementary Information for a reflective display based on the electro-microfluidic assembly of particles within suppressed water-in-oil droplet array [file 41377_2023_1333_MOESM1_ESM.docx]

Supplementary Information for

**A reflective display based on the electro-microfluidic assembly of particles within suppressed water-in-oil droplet array**

Shitao Shen^1,2^, Haoqiang Feng^2^, Yueming Deng^2^, Shuting Xie^2^, Zichuan Yi^3^, Mingliang Jin^2^, Guofu Zhou^2^*, Paul Mulvaney^4^ and Lingling Shui^1,2^*

^1^ Guangdong Basic Research Center of Excellence for Structure and Fundamental Interactions of Matter, Guangdong Provincial Key Laboratory of Nanophotonic Functional Materials and Devices, School of Information and Optoelectronic Science and Engineering, South China Normal University, Guangzhou 510006, China

^2^ International Joint Laboratory of Optofluidic Technology and System (LOTS), National Center for International Research on Green Optoelectronics, South China Academy of Advanced Optoelectronics, South China Normal University, Guangzhou 510006, P. R. China

^3^ School of Electronic Information, University of Electronic Science and Technology of China, Zhongshan Institute, Zhongshan 528402, China

^4^ ARC Centre of Excellence in Exciton Science, School of Chemistry, University of Melbourne, Parkville, VIC, 3010, Australia

*Corresponding author: guofu.zhou@m.scnu.edu.cn and shuill@m.scnu.edu.cn; Tel: +86-20-3931-4813

**The PDF file includes:**

[1. Droplet size and deformation effect 2](#_Toc143181675)

[2. Principle of the particle motion and assembly 3](#_Toc143181676)

[3. Electrohydrodynamic effect in water-in-oil droplet 4](#_Toc143181677)

[4. Pixel closing and opening performance of a droplet array 6](#_Toc143181678)

[5. Optimization of dye concentration 7](#_Toc143181679)

[6. Characterization of the color particles and dye solutions 7](#_Toc143181680)

[7. Details of the COMSOL simulation 8](#_Toc143181681)

[8. Estimating of viewing angle-dependent shifting 10](#_Toc143181682)

## Droplet size and deformation effect

**Figure S1** shows that both the applied electric field and the droplet size influence on the pixel switching performance. In a pixel with the same size, the paired droplet volume plays an important role on the droplet deformation degree, the contact area, and the opening aperture size and ratio. As confined by the sandwich structure of the eMAP, the paired water droplet is compressed into drum-like shape, as illustrated in **Figure S1a**. The contact area of the droplet with the top and bottom surfaces vary with the applied AC field. As *U* increases, the droplet contact area with the top surface changes slightly; while, the bottom contact area increases significantly with *U* due to the dielectrowetting effect[^1^](#_ENREF_1). The contact area ratio is calculated by (the top contact area)/(the bottom contact area). The small droplet demonstrates significant variation of the contact area ratio. While for a large droplet compressed in a pixel well, the contact area ratio is maximized according to the physical confinement of the pixel. The obtained droplet contact area ratio is applied to the COMSOL modeling. With the increase of the droplet size, the droplet deformation degree decreases; while the opening ratio increases under the same electric field. To evaluate the aperture size in the orthogonal direction, the midline length termed as *λ*_x_ and *λ*_y_ are used to denote the opening aperture area along the *x*-axis (parallel to the field direction) and *y*-axis (perpendicular to the field direction). As exhibited in **Figures S1c** and **d** that *λ*_x_ is less sensitive to the droplet size than *λ*_y_. The reason is that the droplet elongation caused by dielectrowetting is mainly along the electric field direction (*x*-axis), while reducing the difference to *λ*_x_ caused by the droplet variation.


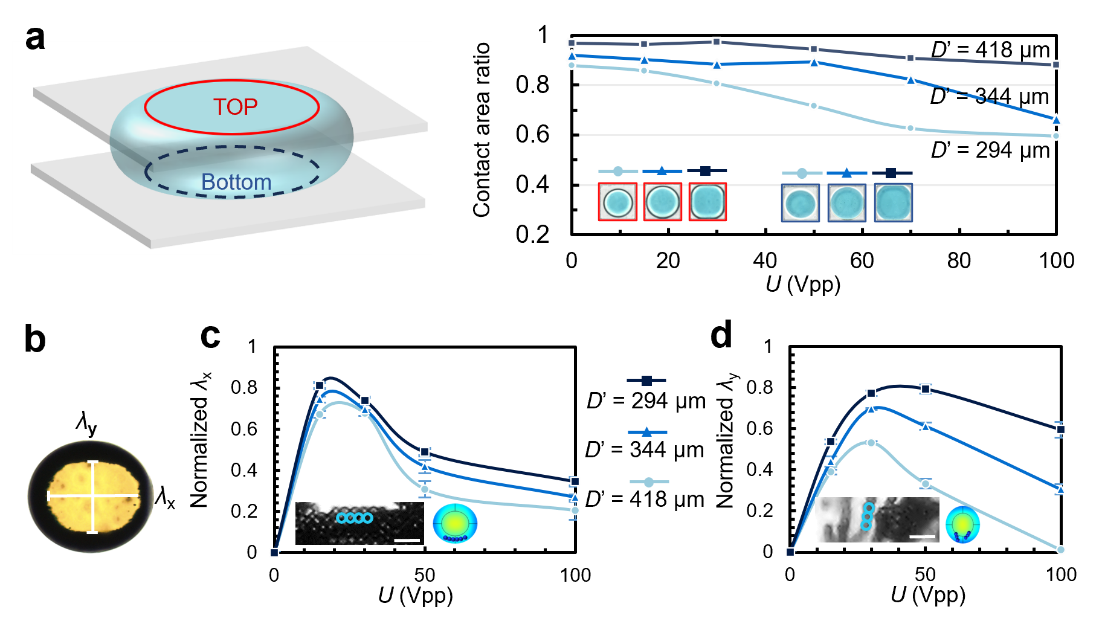


**Figure S1** Droplet deformation and size effect. (**a**) Schematic of the droplet contact with the top (red line) and bottom (blue dotted line) surfaces and the corresponding contact area ratio varying with *U* for different size droplets confined in the same sized pixel well (side length = 345 μm), and *f* = 400 kHz. The droplet size *D*’ is denoted by the diametr of the droplet curvatures analyzed from the topview image. (**b**) A representative droplet pixel with *λ*_x_ and *λ*_y_ denoting the midline length of the aperture area in the *x*-axis and *y*-axis, respectively. (**c**) Normalized *λ*_x_ and (**d**) normalized *λ*_y_ as a function of *U* for the selected three droplets. Insets represent the orientations of the particle assembly structures. The normalized denominator is set to 345 μm. Scale bar: 20 μm.

## Principle of the particle motion and assembly

In addition to the DEP forces, the particle-particle interaction under the electric field plays a role on the switching process. The potential energy of the particle dipole-dipole interaction can be calculated as:

$\text{U}_{\text{d-d}}\text{ = }\frac{\text{1}}{\text{4}\text{π}\text{ε}_{\text{0}}\text{ε}}\text{(}\frac{{\vec{\text{p}}}_{\text{1}}\text{∙}{\vec{\text{p}}}_{\text{2}}\text{-3(}\hat{\text{n}}\text{∙}{\vec{\text{p}}}_{\text{1}}\text{)(}\hat{\text{n}}\text{∙}{\vec{\text{p}}}_{\text{2}}\text{)}}{{\text{|}{\vec{\text{r}}}_{\text{1}}\text{- }{\vec{\text{r}}}_{\text{2}}\text{|}}^{\text{3}}}\text{)}$

where p is the electric dipole moment. Here, we neglect the electric field difference applied to adjacent particles, the dipole moments of adjacent particles can be simlified to:

$\text{p}_{\text{1}}\text{=}\text{ p}_{\text{2}}\text{= p = }{\text{4π}\text{ε}_{\text{m}}\text{f}_{\text{CM}}\text{R}}^{\text{3}}\text{E}$

where the length of vector $\vec{\text{r}}$ corresponds to the distance between the center points, $\hat{\text{n}}$ is the unit vector parallel to the direction vector $\vec{\text{r}}$, and θ is the angle between the vector $\hat{\text{n}}$ and the direction of the electric field. Therefore,

$\text{U}_{\text{d-d}}\text{ = }\frac{\text{p}^{\text{2}}}{\text{4π}\text{ε}_{\text{0}}\text{ε}_{\text{m}}}\left( \frac{\text{1-3}\text{cos}^{\text{2}}\text{θ}}{\text{r}^{\text{3}}} \right)\text{,}$ and $\text{U}_{\text{d-d}}\frac{{\text{f}_{\text{CM}}}^{\text{2}}\text{R}^{\text{6}}\text{E}^{\text{2}}}{\text{r}^{\text{3}}}\text{(1-3}\text{cos}^{\text{2}}\text{θ}\text{)}$

The potential energy shows a positive correlation with the particle radius and is proportional to $\text{E}^{\text{2}}$ and ${\text{f}_{\text{CM}}}^{\text{2}}$. In this way, $\text{f}_{\text{CM}}$ contributes the frequency responsiveness to *U*_d−d_. When *θ* = 54.7º, it can be deduced to

$\text{1 - 3}\text{cos}^{\text{2}}\text{θ}\text{ = 0}$

indicating that *U*_d−d_ reaches zero. If two particles are positioned with *θ* > 54.7º, they will repel to each other; whereas, when *θ* < 54.7º, the attractive interaction will occur. The dipole interaction force can be derivated from *U*_d-d_ as:

$\text{F}_{\text{Dipole}}=\frac{{\text{24π}\text{ε}_{\text{m}}\text{f}_{\text{CM}}}^{\text{2}}\text{R}^{\text{6}}\text{E}^{\text{2}}}{\text{r}^{\text{4}}}$

As shown in **Figure S2a**, when *U* = 10.6 V_rms_ and *f* = 400 kHz (State II), the particles with induced dipole moments parallel to the electric field will form a particle chain mainly along the *x*-direction (in the *x*-*z* plane) near the droplet equator. The dipole interaction force (**F**_dipole_) of the particles is calculated to be ~0.5 pN. While the magnitude of the *z*-component of the dielectrophoretic force (**F**_DEP_) near the droplet equator (*h* = 75 μm) is of ~0.016 pN, which is very close to the particle gravitational force (**F**_gravity_) of 0.017 pN. Thereby, the equator serves as a equilibrium position, the lower and higher positions correspond to the **F**_DEP_ greater and less than **F**_gravity_, respectively (**Figure S2b**).

At State III, the particles distributed nearby the droplet ceiling are subjected to strong interaction under a high amplitude AC voltage (e.g., *U* = 35.4 V_rms_, *f* = 400 kHz). The magnitude of the *z*-component of **F**_DEP_ near the ceiling (~0.066 pN) is higher than that of **F**_gravity_ (0.017 pN), implying that the particles at the droplet ceiling are still subject to a rising dielectrophoretic force. In addition, the magnitude of **F**_dipole_  is ~5.7 pN (**Figure S2c**), which is about 11 times larger than that of State II. Such a strong attractive interaction enables to promote the transition to the “OFF” state of the droplet pixel.


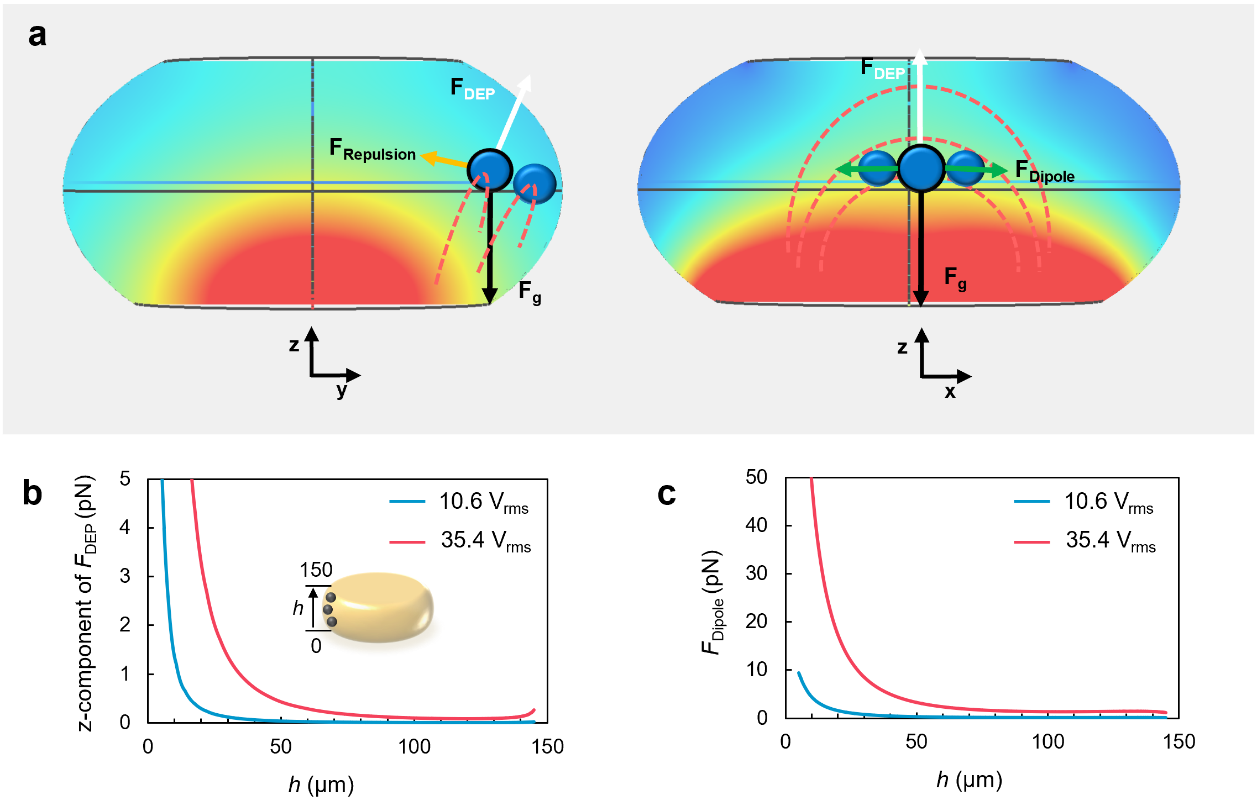


**Figure S2** Particle actuation and motion in droplet. (**a**) Electric field distribution and force analysis at the *y*-*z* (left) and *x*-*z* (right) views of a suppressed droplet pixel at *U* = 10.6 V_rms_ and *f* = 400 kHz. Simulated *z*-component of (**b**) *F*_DEP_  and (**c**) *F*_dipole_ varying with the height (*h*) at *U* = 10.6 and 35.4 V_rms_ (*f* = 400 kHz). The simulation positions at various *h* are near the droplet interface, corresponding to the motion trajectory of the particles.

## Electrohydrodynamic effect in water-in-oil droplet

In our experiments, a flow field can be observed under high amplitude (e.g. ~35.4 V_rms_) and high frequency (e.g. 400 kHz) conditions due to the AC electrothermal (ACET) effect. ACET depends on the temperature gradient induced volume force, which can be calculated from[^2^](#_ENREF_2)

$\text{F}_{\text{ACET}}\text{= }\text{-}\text{M }\text{(}\text{ω}\text{, }\text{T}\text{) (}\frac{\text{εσ}\text{U}_{\text{rms}}^{\text{4}}}{\text{2k}\text{π}^{\text{3}}\text{r}^{\text{3}}\text{T}}\text{)(1-}\frac{\text{2}\beta}{\text{π}}\text{)}\hat{\text{n}_{\beta}}$

where *T* is the temperature, *r* is the distance from any potint to the electrode gap center, and *β* is the angle between the line from the point to electrode gap center and the horizontal direction, and *k* = 0.6 J m^−1^ s^−1^ K^−1^. A dimensionless factor that varies with *f* and *T* can be expressed as:

$\text{M }\text{(}\text{ω}\text{, }\text{T}\text{) = (}\frac{\frac{T}{\text{σ}}\frac{\text{∂}\text{σ}}{\text{∂T}}-\frac{T}{\text{ε}}\frac{\text{∂}\text{ε}}{\text{∂T}}}{1+{(\text{ω}\frac{\text{ε}}{\text{σ}})}^{2}}\text{+}\frac{T}{2\text{ε}}\frac{\text{∂}\text{ε}}{\text{∂T}}\text{)}$

When the magnitude of the Coulomb force equals to the dielectric force, *M* = 0, the cross frequency can be calculated as:

$\text{ω}_{\text{c}}\text{ \textasciitilde}\frac{\text{σ}}{\text{ε}}{\text{(2}\frac{\text{|}\frac{\text{∂}\text{σ}}{\text{σ}\text{∂T}}\text{|}}{\text{|}\frac{\text{∂}\text{ε}}{\text{ε}\text{∂T}}\text{|}}\text{)}}^{\text{0.5}}$

For water, ∂*σ*/*σ*∂*T* = 2% per degree and ∂*ε*/*ε*∂*T* = -0.4%, therefore the magnitude of *f*_c_ = *ω*_c_ (2π)^-1^, which is about 14.2 MHz. The Coulomb force dominates at low frequency (*f* < *f*_c_), and the dielectric force dominates at high frequency (*f* > *f*_c_). Moreover, the Coulomb force at low frequency is about 10 times stronger than the dielectric force at high frequency, the electrothermal effect is thus relatively weak at *f* >14.2 MHz. **Figure S3** shows the simulation results of ACET. At the optimzed *f* = 400 kHz, when *U* increases from 10.6 to 35.4 V_rms_, the ACET effect enhances with the flow velocity magnitude in the range of 1 - 100 μm/s. And the ACET flow velocity in the center and bottom regions is faster than that in the equator region. Furthermore, when the particles locate at a lower plane in the droplet, the DEP effect enhances more rapidly than the ACET effect, and their relative value can be described as:

$\frac{v_{\mathrm{ACET}}}{v_{\mathrm{DEP}}} \text{σ}U^{2}\frac{r^{2}}{R^{2}}$

Therefore, the particles motion near the droplet bottom is mainly attributed to the DEP force, corresponding to the switching from State I to State II. However, when eMAPD switches from State II to State III, the ACET effect at the ceiling region is comparable with the DEP effect. As shown in **Figure S3b**, the flow field in *x-y* plane indicates that the magnitude of ACET flow (~100 μm/s) enables to generate a Stokes force on the particles (**F**_ACET_ ~3.4 pN), which is comparable with the magnitude of dipole interaction force (**F**_dipole_ ~5.7 pN) at the droplet ceiling. Therefore, the ACET flow can affect the formation of State III.


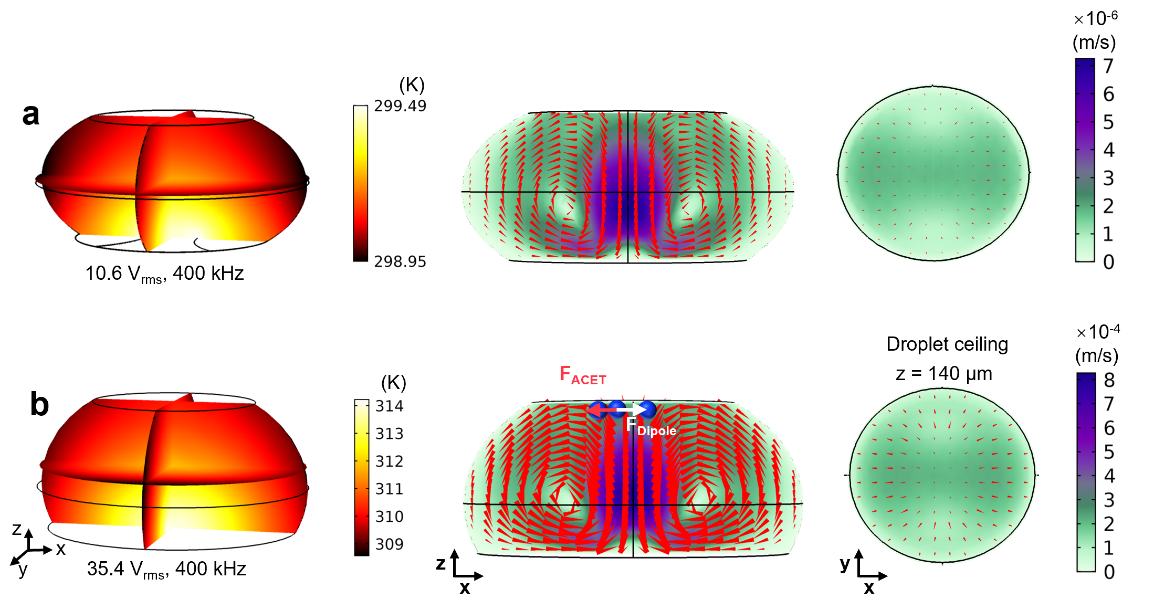


**Figure S3** Simulation of the ACET effect in water-in-oil droplet. The thermal (left) and flow (middle and right) field distributions are simulated at (**a**) *U* = 10.6 V_rms_ and *f* = 400 kHz, and (**b**) *U* = 35.4 V_rms_ and *f* = 400 kHz.

## Pixel closing and opening performance of a droplet array

In the optimized device with the pixel side length of 345 μm, height of 150 μm, and the wall thickness of 20 μm, the obvious color contrast between State I and State II was obtained with properly filled water-in-oil emulsion droplets, as exhibited in **Figure S4**.


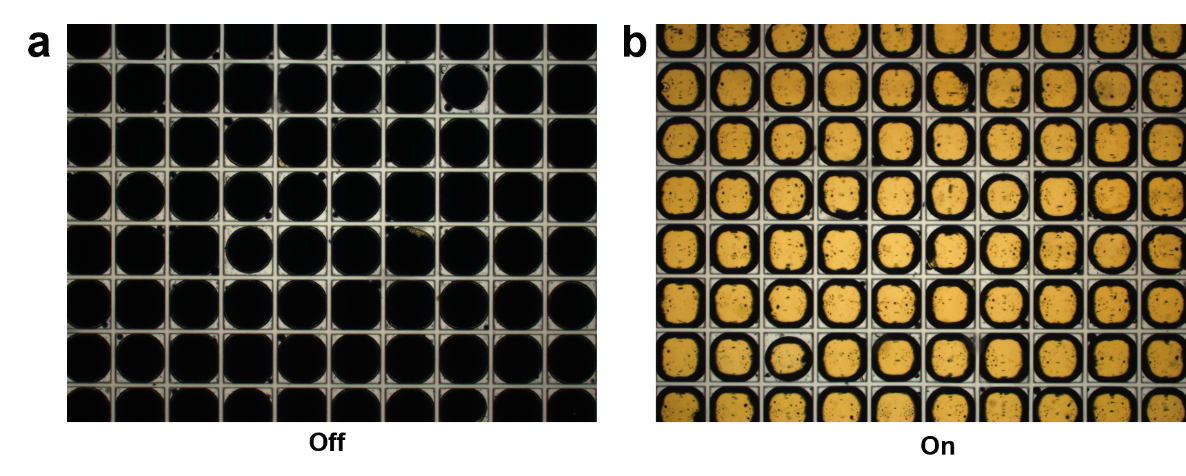


**Figure S4** Switching performance of a droplet pixel array. Optical images showing (**a**) State I at *U* of 0 Vrms and (**b**) State II at 10.6 V_rms_ and 400 kHz in an eMAPD with optimized materials and geometries.

## Optimization of dye concentration

The color brightness and saturation of a display device is dependent on the dye composition and concentration. In general, for the same dye, the high dye concentration corresponds to strong vivid color. However, the increase of concentration would also lead to the increase in water conductivity, resulting in strong electrohydrodynamic flow and low electric field strength. Therefore, we optimized the dye concentration according to the balance of the optical and electrical properties of the dye solution filled device. Taking the yellow dye as the example, as shown in **Figure S5**, with the same gap distance, the reflectivity at 482 nm increases with the dye concentration; while at the same dye concentration, the reflectivity increases with the gap distance. To ensure the DEP effect and meanwhile satisfy the color contrast requirement, the optimized dye concentration of about 0.09 wt% with a conductivity of ~20 mS m^-1^ has been chosen as the aqueous phase. And all other dye concentration optimization is based on the same strategy.


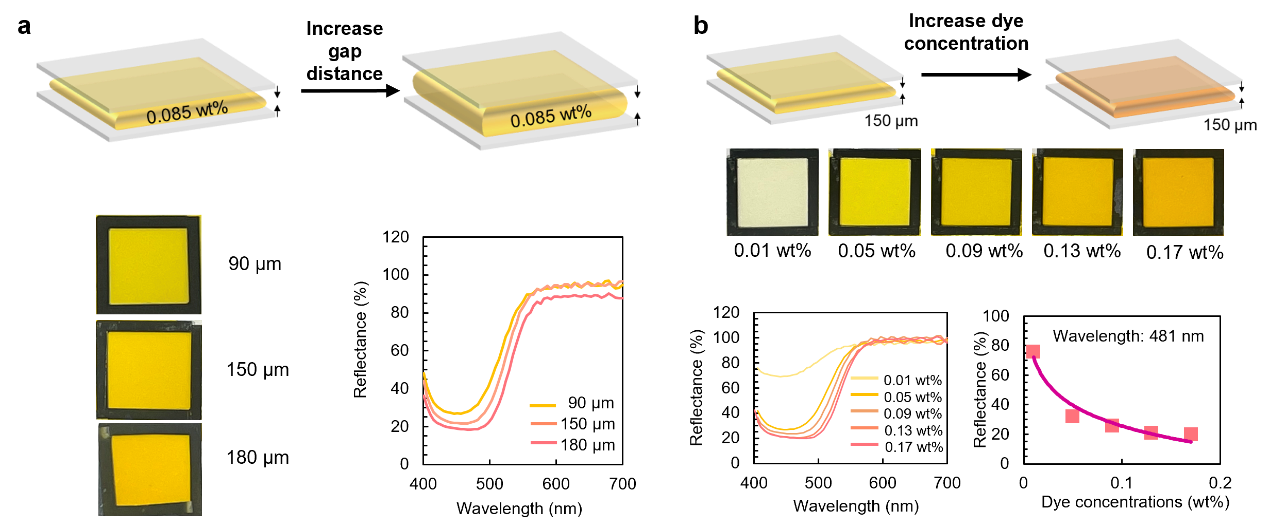


**Figure S5** Optimization of dye concentration. (**a**) Schematic of the yellow dye solution filled devices with different gap distances (top), and the corresponding images and reflection spectra (bottom). The dye weight concentration is fixed at 0.09 wt%. (**b**) Schematic of the devices (gap distance, 150 μm) filled with yellow dye solution at different concentrations (top), and the corresponding images and reflection spectra (bottom).

## Characterization of the color particles and dye solutions

The reflection spectra of particles and dye solutions were also measured and optimized before using in the eMAPD. For the particles, prior to characterization, each type of particle suspension (~12 wt% particles in DI water) was piptted onto a glass slide, and then evaporated at room temperature for 24-48 hours to obtain a particle covered glass sample. The dye solutions were prepared at optimized concentrations, and then filled into a device made of two parallel glass slide separated with a 150 μm thick spacer. The reflection spectra were measured using a fiber-optic spectrometer (USB2000+, Ocean Optics), and the results are shown in **Figure S6**.


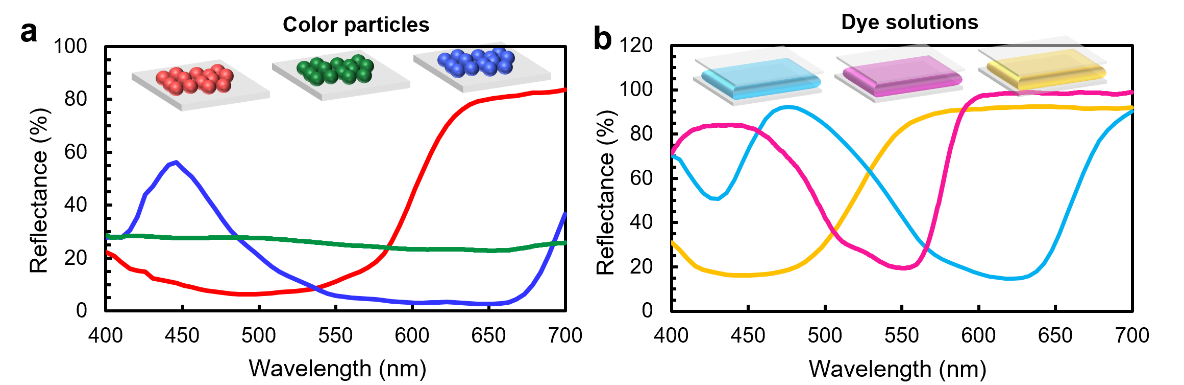


**Figure S6** Characterization of the color particles and dye solution. (**a**) Reflection spectra of the blue, green, and red PS particles. (**b**) Reflection spectra of the cyan, magenta, and yellow dye solutions.

## Details of the COMSOL simulation

A finite element simulation with the electric current model was performed to investigate the electric field distribution using the COMSOL Multiphysics 5.5. The electric potential is assumed to satisfy the Gauss’s law. The frequency-domain analysis is applied, and the electric field calculation combines the time-harmonic equation and the Gauss’s law:

$$\text{}\text{·J = }\text{}\text{·}\left( \text{σ}\text{E}\text{ + }\text{J}_{\text{e}} \right)\text{= -}\text{j}\text{ωρ}$$

$$\text{}\text{·}\text{ε}\text{E}\text{ = }\text{ρ}$$

$$\text{E}\text{ = -}\text{}\text{U}$$

$$\text{U}\text{}\text{ }\text{= }\text{Amplitude }\text{× }\text{cos}\text{(}\text{ωt}\text{)}$$

where *J* and $\text{J}_{\text{e}}$ are the current density and externally generated current density, respectively; $\text{ρ}$ is the space charge density; and *U* is the electric potential. **E** is the electric field, *ω* represents the angular frequency, *t* is the time, *n* denotes the unit normal vector pointing into the electrolyte, and $\text{σ}$ and $\text{ε}$ are the conductivity and permittivity, respectively.

The geometry of the 3D model is built based on the proposed eMAPD device. A box of 1.6×1.6×0.15 mm^3^ is set as the oil phase with a relative permittivity of 2.7 and a conductivity of 1×10^-7^ mS m^-1^. Aqueous droplet is confined to in a cuboid pixel space (345×345×150 μm^3^). The box contains suppressed a drum-like droplet with a long axis of 340 μm and a short axis of 310 μm, corresponding to the results shown in **Figures 2h-l**. The conductivity and relative permittivity of the aqueous droplet are set at 20 mS m^-1^ and 80, respectively. The droplet viscosity, heat capacity and thermal conductivity are set at 1.0×10^-3^ N Sm^-2^, 4.2 kJ (kg K)^–1^ and 0.6 W (m K)^-1^, respectively. The oil heat capacity and thermal conductivity are set at 1.5 kJ (kg K)^–1^ and 0.15 W (m K)^-1^, respectively. The thickness of the hydrophobic layer is set at 140 nm with a relative permittivity of 2.0 and a conductivity of 1.0×10^-7^ mS m^-1^. The mesh of the model consists of triangles (surface) and tetrahedra (volume). The boundary conditions for the electric field are:

in the bulk, $\text{}^{\text{2}}\text{U }\text{= 0}$, $\text{E}\text{ = -}\text{}\text{U}$

at the AC signal applied electrode, $\text{U}\text{ = }\text{Amplitude}\text{ × cos(2}\text{π}\text{f}\text{t}\text{)}$

at the grounded electrodes, $\text{U}\text{ = 0}$ V

and at the wall, $\text{n}\text{·}\text{}\text{U }\text{= 0}$

For the ACET simulation, the initial temperature is set at 298.15 K. The temperature rise can be calculated as:

$\text{}\text{ }\text{c}_{\text{p}}\text{u}\text{·}\text{}\text{T = }\text{k}\text{}^{\text{2}}\text{T}\text{ + }\text{σ}{\text{|}\text{E}\text{|}}^{\text{2}}$

where *T* is the temperature, *c*p is the specific heat, and *k* is the thermal conductivity of the medium. The temperature can induce a flow field in the droplet, and the ACET force per unit volume can be given as[^3^](#_ENREF_3):

$\text{F}_{\text{ACE}}\text{ = }\frac{\text{1}}{\text{2}}\text{R}\text{e}\text{(}\left( \text{α - β} \right)\text{·}\frac{\text{1}}{\text{1+}\left( \text{ω}\text{τ} \right)^{\text{2}}} \text{- }\frac{\text{α}}{\text{2}}\text{) }{\text{|}\text{E}\text{|}}^{\text{2}}\text{ε}\text{}\text{T}$

where $\text{α = }\frac{\text{1}}{\text{σ}}\left( \frac{\text{∂σ}}{\text{∂T}} \right)\text{= -0.004}$ and $\text{β = }\frac{\text{1}}{\text{ε}}\left( \frac{\text{∂}\text{ε}}{\text{∂T}} \right)\text{= 0.02}$. τ = ε/σ with ε and σ the droplet permittivity and conductivity, respectively. In this model, ωτ << 1; therefore, the Columbic force is dominant to the dielectric force.


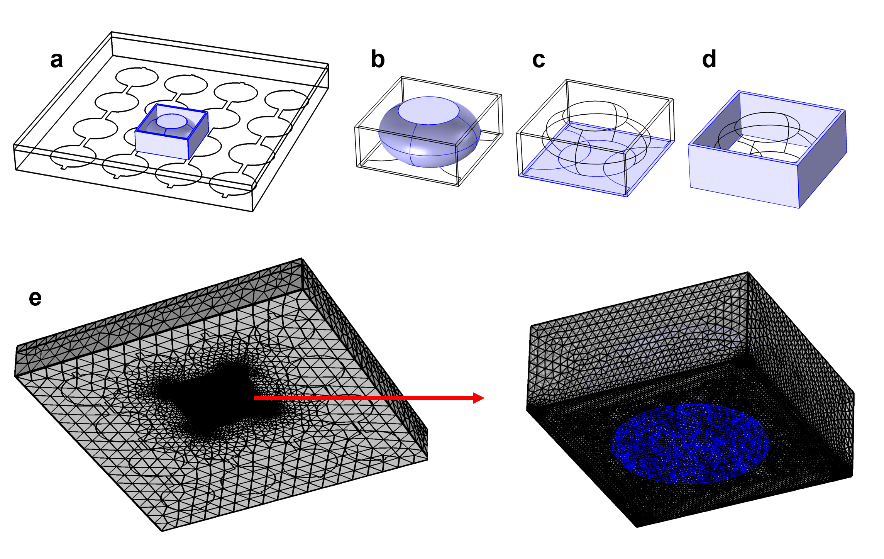


**Figure S7** Details of the COMSOL simulation. Schematic of (**a**) an overall view of an eMAPD with a representative single pixel, (**b**) a droplet in a single pixel, (**c**) the Hyflon layer in a single pixel, and (**d**) the walls of a single pixel. (**e**) Mesh of the eMAPD and pixel model in the simulation.

## Estimating of viewing angle-dependent shifting


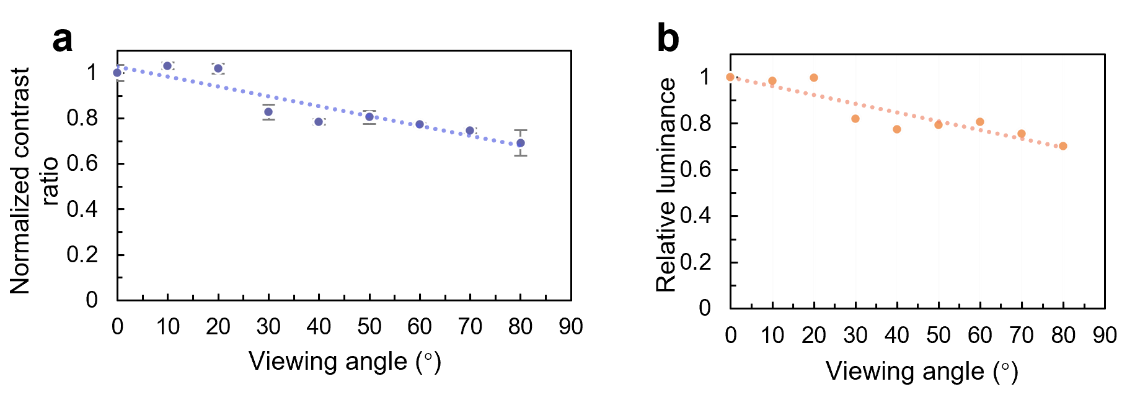


**Figure S8** Viewing angle-dependent shifting in display performance. (**a**) Normalized contrast ratio versus viewing angle. The evaluation of contrast ratio was performed using spectral data at a wavelength of 571 nm. (**b**) Relative luminance as a function of viewing angle.

**References**

1 Edwards, A. M., Brown, C. V., Newton, M. I. & McHale, G. Dielectrowetting: The past, present and future. *Current opinion in colloid & interface science* **36**, 28-36 (2018).

2 Ramos, A., Morgan, H., Green, N. G. & Castellanos, A. Ac electrokinetics: a review of forces in microelectrode structures. *Journal of Physics D: Applied Physics* **31**, 2338 (1998).

3 Wu, Y., Ren, Y. & Jiang, H. Enhanced model‐based design of a high‐throughput three dimensional micromixer driven by alternating‐current electrothermal flow. *Electrophoresis* **38**, 258-269 (2017).
